# *In vitro* and *in silico* methods for the biomechanical assessment of osseointegrated transfemoral prostheses: a systematic review

**DOI:** 10.3389/fbioe.2023.1237919

**Published:** 2023-08-17

**Authors:** Giulia Galteri, Luca Cristofolini

**Affiliations:** Department of Industrial Engineering, School of Engineering and Architecture, Alma Mater Studiorum-University of Bologna, Bologna, Italy

**Keywords:** transfemoral osseointegrated prosthesis, load transfer, mechanical implant stability, stress shielding, stress concentration, *in vitro* mechanical test, *in silico* simulation

## Abstract

The amputee population according to the World-Health-Organization is about 40 million. However, there is a high abandon rate of socket prostheses for the lower limb (25%–57%). The direct connection between the external prosthesis and the patient’s bone makes osseointegrated prostheses for transfemoral amputees advantageous (e.g., improvement of the motor control) compared to socket prostheses, which are currently the gold standard. However, similarly to other uncemented prostheses, the osseointegrated ones are at risk of aseptic loosening and adverse bone remodelling caused by stress-shielding. The preclinical assessment of these prostheses has already been evaluated using different methods which did not provide unanimous and comparable evidence. To compare data from different investigations, a clear and detailed overview of the methods used to assess the performance is necessary. In this review 17 studies investigating the primary stability, stress shielding and stress concentration of osseointegrated transfemoral prostheses are examined. Primary stability consists in the biomechanical stability upon implant insertion. Primary stability is assessed measuring extraction force (either with a pull-out or a push-out test) and micromotion at the interface between the implant and the host bone with LVDT (*in vitro* test) or numerical models. Stress-shielding causes adaptive changes in the bone density around metal implants, and thus in the bone strength and stiffness. Stress-shielding is assessed with strain gauges or numerical models measuring the load transfer and the strain distribution on the surface of the femur, and between the implant and the bone respectively. Stress concentration can lead to the formation of cracks inside the bone, resulting in fractures. The stress concentration is assessed measuring the load transfer and the strain energy density at the interface between the implant and the bone, using numerical models. As a result, a global view and consensus about the methods are missing from all these tests. Indeed, different setup and loading scenario were used in the *in vitro* test, while different model parameters (e.g., bone properties) were used in the numerical models. Once the preclinical assessment method is established, it would be important to define thresholds and acceptance criteria for each of the possible failure scenarios investigated.

## 1 Introduction

According to the World Health Organization, the global population of amputees is about 40 million (Marino et al., 2015; Windrich et al., 2016; Fanciullacci et al., 2021) reported that the lower limb amputation are about 36 million. In the European counties 15 to 30 every 100,000 subjects receive a lower limb amputation every year (Hughes et al., 2020). The leading causes of lower-limb amputation are wartime injury, traumatic events and atherosclerosis (Moxey et al., 2011; Newhall et al., 2016). Approximately 86% of patients with a transfemoral amputation are fitted with a socket prosthesis, which represents the current standard of care (Rommers et al., 1996). However, skin lesions occur in more than 50% of lower limb amputees, resulting in a negative impact on mobility, mechanical stability, and a high abandon rate of use of lower-limb prostheses (25%–57%) (Rommers et al., 1996; Hagberg and Brånemark, 2001; Paternò et al., 2018).

To overcome these problem, osseointegrated prostheses have been developed. In fact, osseointegrated prostheses provide a direct structural connection between the external prosthesis and the remaining living bone. Moreover, these prostheses show many advantages with respect to the socket prostheses, such as an improvement of the motor control, functional capacity and a better hip range of motion (ROM) (Thesleff et al., 2018). Osseointegrated transfemoral prostheses are mainly indicated for adults, generally under 65 years old, with a physically active lifestyle (typical of young patients). These subjects generally expect a significant improvement of the performance, for a long post-operative period. Hence the importance of improving the quality of life of these patients.

Currently commercially available osseointegrated transfemoral prostheses can be divided in two broad categories: the screw-fixation type and the press-fit type. Both types are designed to prevent infections and to achieve the mechanical stability of the implant. However, a correct osseointegration is a challenge, like in other osseointegrated prosthesis, and failure does occur due to septic or aseptic causes (Atallah et al., 2018). The main septic cause is the soft tissue infection at the stoma, and many of the associated consequences are reported in other reviews (Hebert, Rehani, and Stiegelmar 2017; Gerzina et al., 2020). In these reviews, the evolution, clinical outcomes, success rates and complications of different osseointegrated prostheses for amputees are reported, with a follow up greater than 12 months.

The main aseptic mechanical causes of failure are: post-operative periprosthetic fractures, loss of mechanical stability, and failure of the intramedullary stem or of the abutment. Post-operative periprosthetic fractures have an incidence between 3% and 10% (Brånemark et al., 2014; Juhnke et al., 2015; Al Muderis et al., 2016; Örgel et al., 2021)*.* The loss of mechanical stability occur in between 3% and 29% (Roberts et al., 1991; Aschoff and Juhnke, 2016; Muderis et al., 2016; Kagan et al., 2017; Atallah et al., 2018; Hagberg et al., 2020). Other mechanical complications, such as mechanical failure of the intramedullary stem or of the abutment or of the dual cone, occur in between 5% and 30% of patients (Leijendekkers et al., 2017; Brånemark et al., 2019; Ranker et al., 2020; Reetz et al., 2020). Mechanical failures of the implantable components are more likely to occur in the long-term survival of the implant, especially among the more physically active patients (Hagberg et al., 2020; Hagberg et al., 2023).

The methods to investigate such failure scenarios are far from consolidated: quite different methods and different criteria and metrics are reported in the literature. For these reasons, results are difficult to compare or even conflicting. To enable comparing the results from the different investigations, a more systematic understanding of the test and simulation conditions would be important. This would help to improve the pre-clinical assessment of transfemoral osseointegrated prostheses.

Thus, the purpose of this systematic review is to summarize, evaluate and discuss the different methods used for the assessment of primary stability, stress shielding, and stress concentration of the transfemoral osseointegrated prostheses in order to find a systematic approach useful for all those who want to test these loading scenarios.

## 2 Methods

### 2.1 Search strategy

**FIGURE 1:**
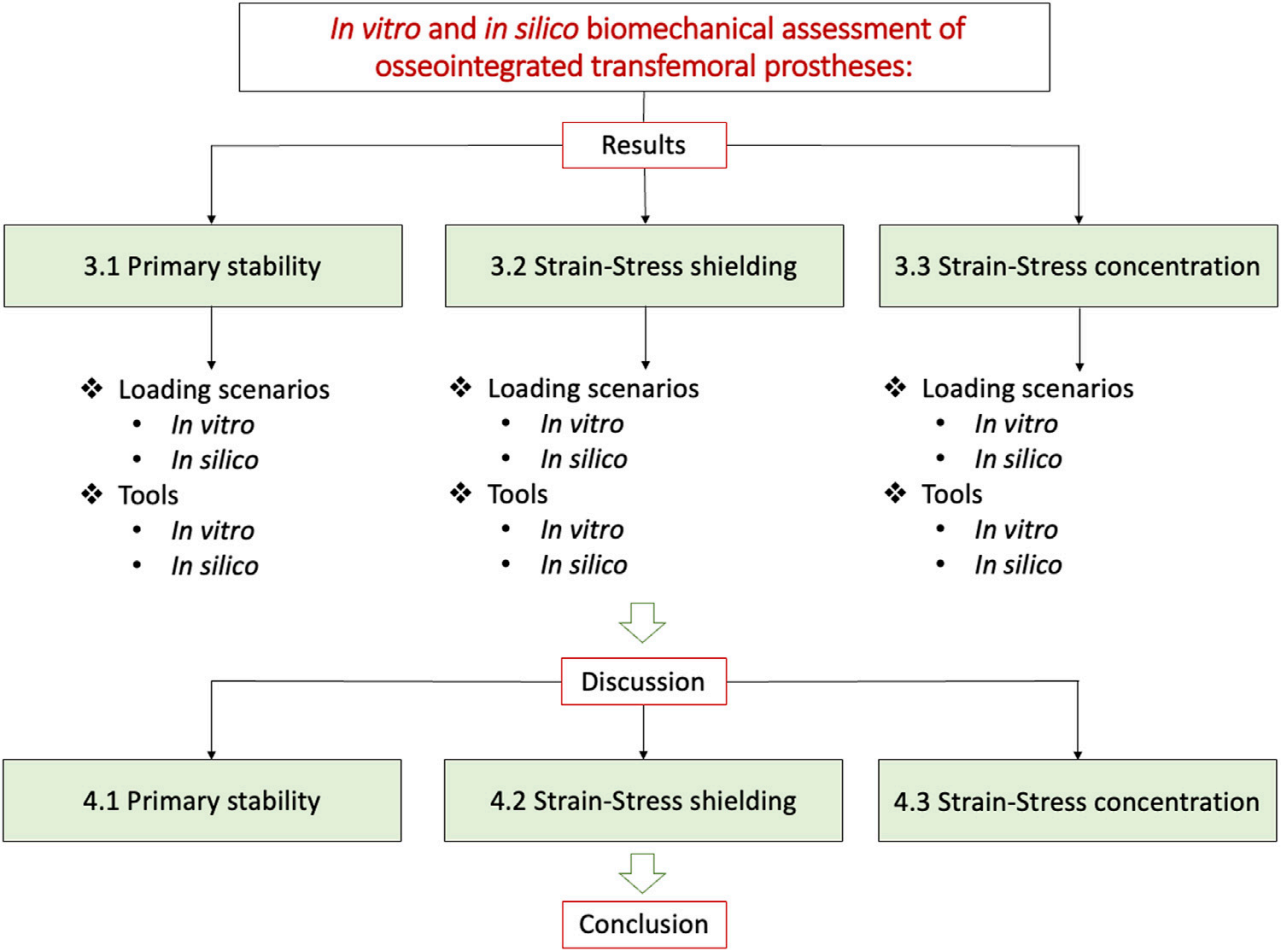
Logic Flow of the systematic review.

The research was performed using the databases: PubMed, ResearchGate, and Google Scholar, covering data before June 2022. PRISMA P guidelines (PRISMA-P, 2022) were followed to build this systematic review, focusing on the published studies of mechanical stability in osseointegrated prostheses for transfemoral amputations (Figure 1). The search strategy was as follow: (Osseointegrated prosthesis OR bone-anchored OR implant OR prosthesis) AND (lower limb OR transfemoral OR distal femur OR femur) AND (primary stability OR stress-shielding OR stress-concentration OR mechanical stability) AND (*in vitro* test OR mechanical test OR *FE model OR* numerical simulation). Results were limited to full-text articles in English. Articles were not restricted based on publication status or date.

### 2.2 Inclusion and exclusion criteria

To be included in the final review, the screened papers had to satisfy the following criteria:

• The first inclusion criterion was the presence of at least one of the search key words in the title and/or in the abstract. The second inclusion criterion was the use of *in vitro* mechanical test or FE models to evaluate the primary stability, the stress-shielding, or the stress concentration, and the failure mode of the osseointegrated transfemoral prostheses.
• The first exclusion criterion were studies about socket prosthesis, upper limb prosthesis and ankle-foot prosthesis. Second exclusion criterion were articles in which the analysis of the primary stability, the stress-shielding, or the stress concentration of the osseointegrated transfemoral prostheses was not reported. Moreover, clinical cohort study and case study were not reported in this review.

### 2.3 Screening methods/studies selection

The authors independently selected and reviewed the article title, key words and abstracts based on the inclusion and exclusion criteria. After the first screening (first exclusion criteria) the authors reviewed the full text articles. The articles that passed the first selection were then fully read. Any disagreements were solved between the reviewers and if something was unclear, the authors of the studies were contacted. The articles found in different databases were included only once. To further expand the research, citations of the papers and related articles were also tracked.

### 2.4 Logic flow of the systematic review

This systematic review aimed to summarize, evaluate, and discuss the different *in vitro* and *in silico* methods used for the biomechanical assessment of an osseointegrated transfemoral prosthesis (Figure 1). Thus, the next paragraphs are organized to present the methods related to the main three modes of biomechanical failure described in the Introduction: the lack of implant stability (Section 3.1), the strain/stress shielding (Section 3.2) and the strain/stress concentration (Section 3.3). Each of these topics will be analyzed and discussed in a similar by evaluating:

• The loading scenarios used to test each mode of failure, in terms of the loading conditions simulated either in the experimental test, or in the *in silico* model.
• Measured parameters and tools used either experimentally, or to implement the *in silico* models.

## 3 Results

**FIGURE 2:**
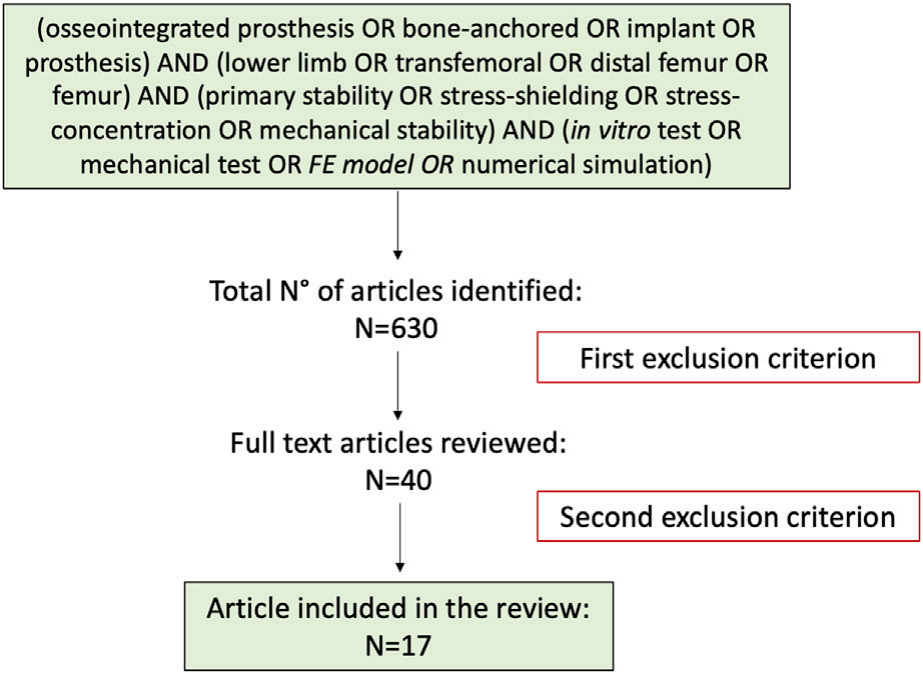
Search strategy and overview of search results.

A total of 630 articles were identified in the initial search process. Following the screening of the titles and abstract and after the exclusion of duplicates articles, 590 studies were excluded, and 40 studies remained for full-text evaluation (first exclusion criterion). After the full-text evaluation, 17 articles were included in this review (second exclusion criterion). The PRISMA flowchart for the study is reported in Figure 2. A recent review (Thesleff et al., 2018) covered specifically the constructive details of the existing osseointegrated prostheses. That review provided an overview of the devices currently available on the market in terms of different materials, coatings, modularity. Therefore, constructive details are not further discussed in the present review.

The papers included in this review use different terms to indicate the osseointegrated prosthesis. This review uses standardized nomenclature for comparison of data from different works. In particular:

• Osseointegrated limb prosthesis, percutaneous implant, intraosseous transcutaneous amputation prosthesis, bone anchored prostheses in this review, for consistency, will always be called “osseointegrated lower-limb prosthesis.”
• Osseointegrated prostheses for transfemoral amputees in this review will always be called “Transfemoral Osseointegrated Implant (TOI).”

### 3.1 Methods for the evaluation of the primary stability

Primary stability is defined as the biomechanical stability upon implant insertion, and it is crucial to achieve the long-term stability. Primary stability is mainly achieved when interface micromotion does not exceed the threshold for granting bone ingrowth and osseointegration. There is a general consensus about a range of micromotions that can be tolerated: between 20 and 150 μm (Pilliar, Lee, and Maniatopoulos, 1986; Kohli, Stoddart, and van Arkel, 2021). In fact, an excessive interface micromotion can interfere with the process of osseointegration, leading to formation of a fibrous tissue layer at the implant-bone interface. Thus, this negative self-sustained loop can lead to aseptic loosening (i.e., loss of primary stability of the implant within the bone, with no sign of sepsis) (Cameron, Pilliar, and Macnab, 1973; Pilliar, Lee, and Maniatopoulos, 1986; Fukuoka, Yoshida, and Yamano, 2000; Duyck et al., 2006; Winter, Klein, and Karl, 2012). Since micromotion can interfere with the process of osseointegration (Winter, Klein, and Karl, 2012), the mechanical test evaluating micromotion is crucial to assess the osseointegration process. Micromotion can be classified as permanent migrations and inducible micromotions:

• Permanent migrations are defined as the non-reversible implant motion that is accumulated cycle after cycle, that remains even after removing the load. Permanent migrations do not necessarily prevent implant stability, in case they settle over time. The main shortcoming of permanent migrations (if not accompanied by excessive inducible micromotions, see below) is that they may result in a final implant position different from the intended one.
• Inducible (or elastic) micromotions are defined as a temporary relative motion between the prosthesis and the host bone that is reversing after removing the load (Cristofolini et al., 2003). Excessive inducible micromotions can cause pain, mechanical instability and fibrous tissue formations.

Primary stability can be assessed with different methods, such as measuring the extraction force (pull-out or push-out tests) or the micromotions at the interface between the implant and the host bone. Since a standard procedure to test and assess the primary stability of osseointegrated prostheses does not exist, different methods were developed to measure the pull-out or push-out load and the micromotion.

Experimental *in vitro* pull-out and push-out tests were performed at the time of *in vitro* implantation (Welke et al., 2013; Jeyapalina et al., 2014; Barnes et al., 2019) and after 3-6-9-12 months *ex vivo* on sheep (Jeyapalina et al., 2014). Micromotions were investigated both *in vitro* at the time of implantation (Barnes et al., 2019), and through *in silico* simulated rehabilitation exercises (Prochor and Anna Mierzejewska, 2019).

#### 3.1.1 Loading scenarios

**FIGURE 3:**
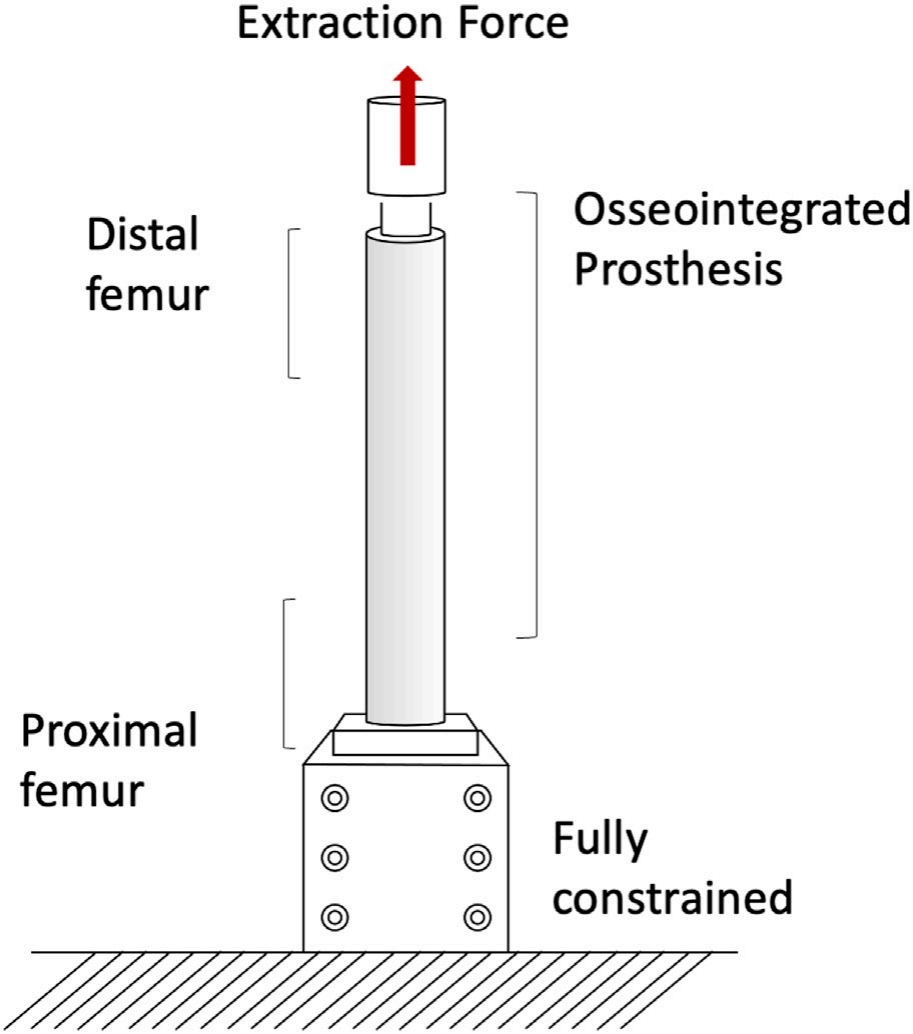
Generic experimental setup for the pull-out tests.

The pull-out and push-out tests allow to assess how strong and well anchored the implant is under extremely simplified loading conditions. Pull-out tests were performed by applying a tensile force to the prostheses until the extraction of the prosthesis or the failure of the bone (A generic experimental setup is represented in Figure 3) (Welke et al., 2013; Jeyapalina et al., 2014). The proximal femur was embedded and clamped (ensuring axial alignment) (Welke et al., 2013; Jeyapalina et al., 2014). To grip the prosthesis a hole was drilled in the distal part of the prosthesis and a stainless-steel rod was placed through hole and connected to the actuator (Jeyapalina et al., 2014) or to a universal-joint (Welke et al., 2013). Also push-out tests can be used, in case of very short stems. To push-out the prosthesis a rod was attached to the actuator and pushed the implant out of the canal of a short segment resected from the femur (Barnes et al., 2019). Both in the pull-out and push-out tests, the specimens were loaded in displacement control with a constant rate of a range between 5 mm/min and 100 mm/min (Welke et al., 2013; Jeyapalina et al., 2014; Barnes et al., 2019) until ultimate failure (loads and methods are summarized in Table 1).

**TABLE 1:** Methods to assess the primary stability are reported.

Author	Tools	Specimens	Femur geometry	Bone properties	Stem properties	Interface	Preload	Loading scenario	Testing protocol	Main outcomes
Welke et al. (2013)	Uniaxial material testing system	Composite femurs (*n* = 8)	N.A.	N.A.	Ti6Al4V stem E = 110 GPa, v = 0.33	N.A.	N.A.	Axial pull-out	Tensile loading to failure 6 mm/min	Max. Force AVERAGE± SD (3,571 ± 919) N
Jeyapalina et al. (2014)	Uniaxial material testing system	Sheep femurs (*n* = 32)	N.A.	N.A.	Ti6Al4V stem E = 115 GPa v = 0.30	N.A.	N.A.	Axial pull-out	Tensile loading to failure 100 mm/min	Max. Force AVERAGE ±SD (988 ± 847) N at time 0, (13,485 ± 1,626) N after 12 months
Barnes et al. (2019)	Uniaxial material testing system	Cadaveric human femurs (*n* = 9)	N.A.	N.A.	- Stainless steel stem - Stiffness matched stem	N.A.	N.A.	Axial push-out	Tensile loading to failure 5 mm/min	Max. Force AVERAGE ±SD (2,248 ± 398) N
Barnes et al. (2019)	LVDT	Cadaveric human femurs (*n* = 9)	N.A.	N.A.	- Stainless steel stem - Stiffness matched stem	N.A.	N.A.	Cyclic compression and bending load	100 cycles at 1 Hz varying loading regime: 0–1.4 BW with R-ratio = 0.1	Inducible micromotion <10 μm
Prochor and Anna Mierzejewska (2019)	Generic *in silico* model	Human femur	From CT scan	Isotropic cortical bone (E = 20 Gpa, v = 0.3) Trabecular bone (E = 0.96 Gpa,v = 0.12)	- PEEK stem E = 12.50 GPa v = 0.40 - Ti6Al4V stem E = 110 GPa, v = 0.33	Friction coefficient = 0.4	N.A.	Static compression	Monotonic Axial load (force up to 1,000N)	Permanent migrations <50 μm

Note: E = Young’s Modulus, G = Shear modulus, v = Poisson’s ratio, *ρ* = ash density, Fr= Resultant Force, Mr = Resultant Moment.

Column 2 indicates the tools used to measure the primary stability. Column 3 to 7 indicate the characteristics of the *in silico* model or *in vitro* experimental tests, while in the following columns were summarized the loading scenario and the testing protocol. The last one summarizes the main outcomes.

**FIGURE 4:**
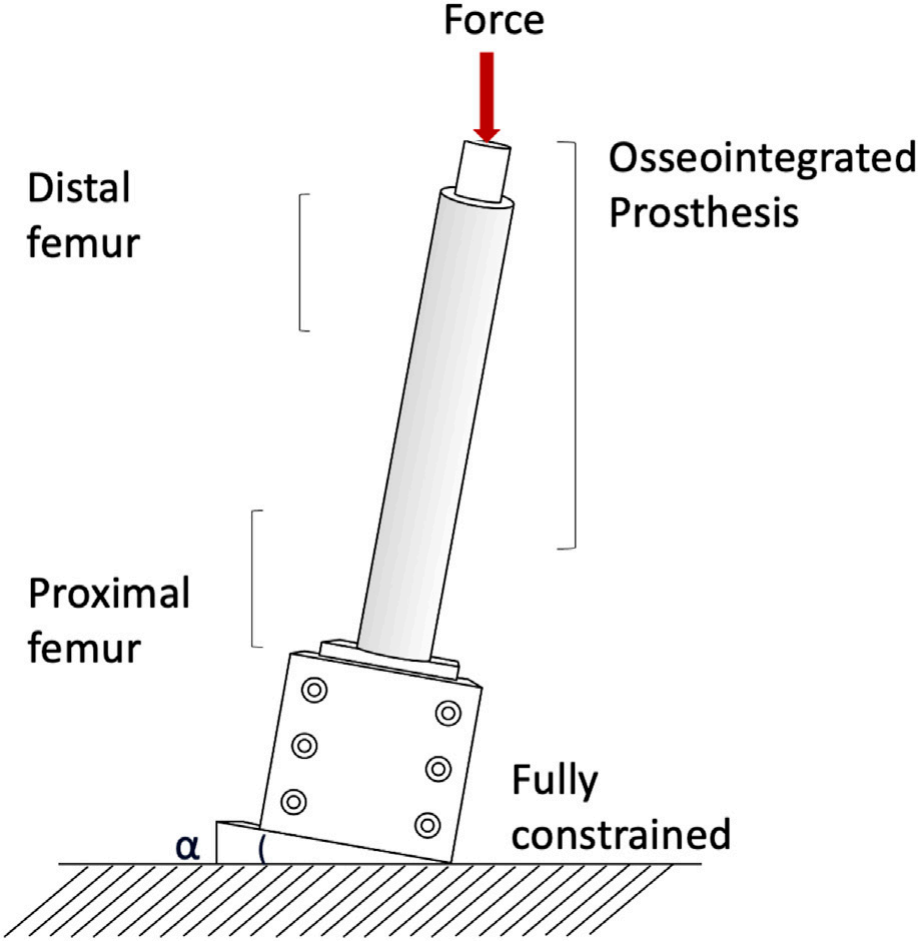
Generic setup of the experimental mechanical test used to assess the stress shielding and stress concentration.

Measuring permanent migrations and inducible micromotions is useful to assess whether the prosthesis has mobilized during the movement and involves more complex experimental setups or numerical simulation. Prochor et al. simulated with a numerical model a static load bearing exercise (Prochor and Anna Mierzejewska, 2019). A static axial force of 1000 N was applied simulating a patient with a body weight (BW) of approximately 100 kg (Prochor and Anna Mierzejewska, 2019). A more complex experimental loading scenario has been designed by (Barnes et al., 2019) to measure inducible micromotions of the prostheses. Specimens were tilted and aligned following a modified version of the international society of Biomechanics (Wu et al., 2002). Thanks to that, the specimens were subjected to a combination of compression and bending moment, simulating physiological conditions (a generic experimental setup is represented in Figure 4). An increasing cyclic load until 1.4 of the body weight (BW) at 1 Hz was applied to measure the inducible micromotion of the prosthesis from the last 90 cycles (Barnes et al., 2019). Loads and methods are reported in Table 1.

#### 3.1.2 Tools

Both the pull-out and push-out extraction force was measured with a uniaxial material testing system (Jeyapalina et al., 2014; Barnes et al., 2019). The applied force was measured directly by the load cell of the testing machine. In some case, the interface shear strength was computed by dividing the applied force by the interface surface area (Barnes et al., 2019).

The *in silico* model was based on a human femur CT scan. The friction coefficient at the interface between the simulated implant and the bone must reflect the immediate post-operative conditions. A coefficient of friction of 0.4 was considered by (Prochor and Anna Mierzejewska, 2019). Moreover, to simulate continuous bone remodeling around the implant, a suitable Fortran subroutine was used during the calculation process in which, for each calculation step, the density and Young’s modulus of bone tissues were updated in each finite element. The initial values of bone Young’s modulus of cortical bone and trabecular bone were respectively 20 GPa and 0.96 GPa. Poisson’s coefficient was assumed to be constant for both bone tissues throughout the simulation. The threshold values for bone density were 𝜌max = 2.000 g/cm^3^ and 𝜌min = 0.200 g/cm^3^ (Prochor and Anna Mierzejewska, 2019).

In the *in vitro* experimental tests, the inducible micromotion of the implant in the host bone was measured with linear variable differential transformers (LVDT) (Barnes et al., 2019). A combination of three LVDTs and a ring was used to measure micromotions in the axial direction and in bending.

### 3.2 Methods for the evaluation of the strain-stress shielding

Stress shielding is a phenomenon that causes adaptive changes in bone morphology, and in bone tissue strength and stiffness in response to the altered mechanical environment caused by the presence of metal implants. Adverse bone remodelling leads to a lack of bone stock in case of surgery revision, weakens the bone, and contributes to implant loosening (Kagan et al., 2017; L Cristofolini et al., 2009). To the authors’ knowledge, only few *in vitro* studies were performed to evaluate the load distribution and the subsequent strain shielding of an osseointegrated transfemoral prosthesis (Cristofolini et al., 1998; Tomaszewski et al., 2013; Ahmed et al., 2020). In fact, the stress and strain of the bone surrounding the implant are difficult to measure *in vitro* and nearly impossible to measure *in vivo.* Therefore, *in silico* finite element models can advantageously be used to generate these information (Xu et al., 2006; Tomaszewski et al., 2010; Tomaszewski et al., 2012a; Tomaszewski, et al., 2012b; Newcombe et al., 2013; Tomaszewski et al., 2013; Stenlund et al., 2017; Prochor and Sajewicz, 2018; Prochor and Anna Mierzejewska, 2019).

#### 3.2.1 Loading scenarios

Both *in vitro* and *in silico* studies simulated specific loading cases from a normal walking cycle: at the heel strike (25%) (Cristofolini et al., 1998; Xu et al., 2006; Tomaszewski et al., 2013; 2010; Tomaszewski et al., 2012a; Tomaszewski et al., 2012b; Newcombe et al., 2013; Prochor and Sajewicz, 2018; Prochor and Sajewicz, 2019; Prochor and Anna Mierzejewska, 2019) and toe-off (55%) (Tomaszewski et al., 2013; 2010; Tomaszewski et al., 2012a; Tomaszewski et al., 2012b; Newcombe et al., 2013; Prochor and Sajewicz, 2018; Prochor and Sajewicz, 2019; Prochor and Anna Mierzejewska, 2019). Tomaszewski et al. also considered a one leg stance (Tomaszewski et al., 2013) and a forward fall loading condition (Tomaszewski et al., 2012a).

Both in the *in vitro* and in the *in silico* simulations, one end of the femur (usually the proximal one, away from the prosthetic tip to avoid artifacts in the peri-prosthetic region due to the boundary conditions) was fully constrained, and loads were applied to the other end (usually the distal one, through the implanted prosthesis) (A generic experimental setup is represented in Figure 4).

In the *in vitro* experiment, two configurations were tested: axial compression (Cristofolini et al., 1998; Tomaszewski et al., 2013), and a medio-lateral bending moment (in this case, the femur was tilted and aligned in order to apply a combination of compression and bending moment, simulating physiological conditions) (Cristofolini et al., 1998; Tomaszewski et al., 2013; Ahmed et al., 2020) (loads and methods are summarized in Table 2).

**TABLE 2:** Methods to assess the stress shielding are reported.

Author	Tools	Specimens	Femur geometry	Bone properties	Stem properties	Interface	Preload	Loading scenario	Testing protocol	Outcomes
Cristofolini et al. (1998)	Strain gauges	Composite femur (×4)	N.A.	N.A.	COMPRESS Spring- preloaded Titanium stem E = 110 GPa	N.A.	20 cycles at 1,200 N	1) Cyclic compression 2) Cyclic Compression and Bending load	1) F = 1,000 N	Method to assess the strains of osseointegrated transfemoral prostheses
									2) 19 Nm	
Tomaszewski et al. (2013)	Strain gauges	Human cadaveric femur (×7)	N.A.	N.A.	- OPRA E = 110 GPa v = 0.3 - Ti6Al4V stem and outer part PEEK	N.A.	N.A.	1) heel strike (25% gait cycle)	1)F = 805 N	More stress shielding in the distal region (typical Gruen zones 1 and 7). Lower strains during toe off
								2) toe off (55% gait cycle)	2) F = 720 N	
								3) one leg stance	3) F = 800 N	
Ahmed et al. (2020)	DIC (displacement) and Strain gauges (strains)	Human cadaveric femur (×1)	N.A.	N.A.	Ti6Al4V stem E = 115 GPa v = 0.30	N.A.	100 N	Incremental loads (as a multiple of BW) with loading and unloading step	F = 280–2,949 N	Validation of a *in silico* model
Ahmed et al. (2020)	Generic *in silico* model	Human cadaveric femur	From CT scan (slice thickness 0.6 mm)	Orthotropic cortical bone E_X_ = 12.00 GPa, E_Y_ = 20.00 GPa, E_Z_ = 13.40 GPa, v_XY_ = 0.22, v_YZ_ = 0.35, v_XZ_ = 0.38, G_XY_ = 5.61 GPa, G_YZ_ = 6.23 GPa, G_XZ_ = 4.53 GPa	Ti6Al4V stem. E = 115 GPa v = 0.30	Friction coefficient = 0.4	N.A.	1) Early stance (from Orthoload)	1) Fr = 183 N	Stem stiffness affects predicted bone loss. Bone resorption decreased in the more flexible stemmed model
								2) Early stance (from Orthoload) with different alignment (difference between femur and stem)	2) Fr = 2,120 N	
Tomaszewski et al. (2010)	Generic *in silico* model	Human femur	From CT scan (slice thickness 3 mm)	E derived from *ρ*. Cortical bone E = 10,200 *ρ*. Trabecular bone E = 33,900 *ρ*. Transition E = 5,307 *ρ* +469. v = 0.35	- ISP stem (cob-chro-mol) E = 210 GPa, ISP layer (spongiosa metal) E = 1 Gpa, - OPRA E = 110 GPa. v = 0.3	Two Friction coefficient conditions - 0.4 - Implant and bone bonded	N.A.	1) heel stryke (25% gait cycle)	1) Fr = 787 N, Mr = 32 Nm	More stress shielding in the distal region (close to the osteotomy. typical Gruen zones 1 and 7)
								2) toe off (55% gait cycle)	2) Fr = 220 N, Mr = 38 Nm	
Tomaszewski et al. (2012b)	Generic *in silico* model	Human femur	From CT scan (slice thickness 3 mm) T score 0.1	E derived from *ρ*. Cortical bone E = 10,200 *ρ* Trabecular bone E = 33,900 *ρ*. Transition E = 5,307 *ρ* +469. v = 0.4	- ISP stem (cobalt-chro alloy) E = 210 GPa, 2) Ti6Al4V E = 114 GPa, - OPRA E = 110 GPa v = 0.3	friction coefficient. between stem and sleeve = 0.1. Implant and bone were bonded	N.A.	1) heel strike (25% gait cycle)	1) Fr = 787 N, Mr = 32 Nm	Bone loss in the distal region (Gruen zones 1 and 7)
								2) toe off (55% gait cycle)	2) Fr = 220 N, Mr = 38 Nm	
								3) forward fall loading	3) Fr = 678 N, Mr = 103 Nm	
Tomaszewski et al. (2012a)	Generic *in silico* model	Human femur	From CT scan (slice thickness 3 mm) T score 0.1	E derived from *ρ*. Cortical bone E = 10,200 *ρ*. Trabecular bone E = 33,900 *ρ*. Transition E = 5,307 *ρ* +469. v = 0.35	- ISP stem (cob-chro-mol) E = 210 GPa, ISP layer (spongiosa metal) E = 1 GPa, - OPRA E = 110 Gpa. v = 0.3	Implant and bone were bonded	N.A.	1) heel strike (25% gait cycle)	1) Fr = 787 N, Mr = 32 Nm	Bone loss in the distal region (Gruen zones 1 and 7)
								2) toe off (55% gait cycle)	2) Fr = 220 N, Mr = 38 Nm	
Newcombe et al. (2013)	Generic *in silico* model	Human cadaveric femur	From CT scan (slice thickness 2 mm)	The values are reported in Table 1 of the paper	Ti6Al4V stem E = 110 GPa, v = 0.3	Implant and bone were bonded	N.A.	1) Bending moment	1) 143 N	Stress shielding adjacent to the implant
								2) axial load	2) 664 N	
								3) axial torque	3) 8 Nm	
Prochor and Anna Mierzejewska (2019)	Generic *in silico* model	Human femur	From CT scan	Isotropic cortical bone E = 20 GPa, v = 0.3 Trabecular bone E = 0.96 GPa v = 0.12	- PEEK stem E = 12.50 GPa v = 0.40 - Ti6Al4V stem E = 110 GPa, v = 0.33	Friction coefficient = 0.4	N.A.	1) heel strike (25% gait cycle)	1) Fr = 787 N, Mr = 32 Nm	Bone loss in the distal region (typical Gruen zones 1 and 7). Smaller implant diameter showed a reduction of the stress shielding
								2) toe off (55% gait cycle)	2) Fr = 220 N, Mr = 38 Nm	
Stenlund et al. (2017)	Generic *in silico* model	N.A.	Cylindrical geometry	Anisotropic longitudinally E = 16.7 GPa, Transversely E = 11.5 GPa. v = 0.3	Ti6Al4V stem E = 110 GPa. v = 0.35	-Friction coefficient = 0.2 between the transplanted bone and the abutment - Friction coefficient = 0.35 between the abutment and the implant	N.A.	Gait cycle	F = 831 N M = 39.4 Nm	Bone loss in the distal region. Interface stresses Tensional (1.8–11.1) MPa Compressive (9.6–12.5) MPa Shear stress (5.4–6.4) MPa
Xu et al. (2006)	Generic *in silico* model	Human femur	From CT scan	Transversely Isotropic Longitudinal E = 18 GPa Radial and circumferential E = 13 GPa G = 7 GPa v = 0.3	Titanium stem E = 115 GPa, v = 0.30	Implant and bone bonded	N.A.	heel stryke (25% gait cycle)	Fr = 3,750 N Mr = 75 Nm	Smaller implant showed a reduction of the stress shielding effect

Note: E = Young’s Modulus, G = Shear modulus, v = Poisson’s ratio, *ρ* = ash density, Fr= Resultant Force, Mr = Resultant Moment.

Column 2 indicates the tools used to assess the stress shielding. Column 3 to 7 indicate the characteristics of the *in silico* model or *in vitro* experimental tests, while in the following columns were summarized the loading scenario and the testing protocol. The last one summarizes the main outcomes.

In all the *in silico* simulations different loads taken from the experimental measurements (Lee et al., 2008a; Stenlund et al., 2017) and from Orthoload were applied at the end of the femur (Xu et al., 2006; Tomaszewski et al., 2010; Tomaszewski et al. 2012a; Tomaszewski et al. 2012b; Newcombe et al., 2013; Stenlund et al., 2017; Prochor and Sajewicz, 2018; Prochor and Anna Mierzejewska, 2019; Ahmed et al., 2020) (loads and methods are summarized in Table 2).

#### 3.2.2 Tools

In the *in vitro* experiments, both composite femur (Cristofolini et al., 1998) and human cadaveric ones (Tomaszewski et al., 2013; Ahmed et al., 2020) were used. Strain gauges were used to measure the strain magnitude at selected locations on the femur surface, before (Cristofolini et al., 1998) and after implantation (Cristofolini et al., 1998; Tomaszewski et al., 2013; Ahmed et al., 2020).

In the *in silico* simulations (Tomaszewski et al., 2010; Tomaszewski et al. 2012a; Tomaszewski et al. 2012b; Prochor and Anna Mierzejewska, 2019), derived the Young’s moduli of the bone elements from ash densities, while (Xu et al., 2006; Newcombe et al., 2013; Stenlund et al., 2017; Prochor and Sajewicz, 2018; Prochor and Anna Mierzejewska, 2019; Ahmed et al., 2020) considered the Young’s moduli of the bone elements homogeneous. In some models, to reflect the immediate post-operative condition, an interface contact with a coefficient of friction between 0.2 and 0.4 were used (Tomaszewski et al., 2010; Stenlund et al., 2017; Prochor and Sajewicz, 2019; Prochor and Anna Mierzejewska, 2019; Ahmed et al., 2020). In other cases, a fully bonded interface was simulated to represent ideal interface bonding (Xu et al., 2006; Tomaszewski et al., 2012a; Tomaszewski et al., 2012b; Newcombe et al., 2013; Newcombe et al., 2013; Stenlund et al., 2017; Prochor and Sajewicz, 2018) assessed the load distribution in a specific load condition. Instead, to assess the consequences of strain-stress shielding, the strain energy density threshold was used as the stimulus for adaptive bone remodeling (Xu et al., 2006; Tomaszewski et al., 2010; Tomaszewski et al. 2012a; Tomaszewski et al. 2012b; Prochor and Sajewicz, 2019; Ahmed et al., 2020).

### 3.3 Methods for the evaluation of the stress concentration and fracture

Post-operative periprosthetic fractures are most often caused by stress concentrations and occur when the patient abruptly changes the walking speed during gait (Juhnke et al., 2015; Al Muderis et al., 2016; Örgel et al., 2021; R; Brånemark et al., 2014). For this reason, the stress concentration tests focused on the ultimate load of the implanted femur (Welke et al., 2013) and on the stress concentration around the implant during daily activities (Ming, Xiang, and Yubo, 2005; Xu et al., 2006; Lee, et al., 2008b; Helgason et al., 2009; Tomaszewski et al., 2013; 2010; Tomaszewski et al., 2012b; Newcombe et al., 2013).

#### 3.3.1 Loading scenarios

The risk of failure of the osseointegrated lower limb prostheses was evaluated when the prosthesis was subject to a pure bending moment, focusing on the most critical load under simplified loading scenario (Welke et al., 2013). The specimens were mounted in a custom four-point bending device on the material testing machine, applying a bending moment in anterior-posterior direction until failure (Welke et al., 2013). Tomaszewski et al. (2013) simulated specific loading cases from a normal walking cycle: at the heel strike (25%), at the toe-off (55%), and a one leg stance. One end of the specimens was fully constrained, and loads were applied to the other end. In order to simulate physiological conditions, the femur was tilted and aligned (Figure 4). Thanks to that, the specimen was subjected to a combination of compression and bending moment (Tomaszewski et al., 2013).

An *in silico* model was developed to simulate the maximum loads experienced during normal daily activities (Newcombe et al., 2013). The loading conditions (bending moment, axial force and axial torque) were applied individually as three different loading conditions, in order to investigate the effects of each component of load separately (Newcombe et al., 2013). A more complex loading scenarios have been designed, to simulate selected loading cases from a normal walking cycle at the heel strike (25%) (Xu et al., 2006; Helgason et al., 2009; Tomaszewski et al., 2010; Tomaszewski et al., 2012a) and the toe-off (55%) (Lee, et al., 2008b; Tomaszewski et al., 2010; Tomaszewski et al., 2012b), and forward fall loading (Tomaszewski et al., 2012a). The load values are summarized in Table 3.

**TABLE 3:** Methods to assess the stress concentration are reported.

Author	Tools	Specimens	Femur geometry	Bone properties	Stem properties	Interface		Preload	Loading scenario	Testing protocol	Outcomes
Welke et al. (2013)	Uniaxial material testing system	Human cadaveric femur	N.A.	N.A.	N.A.	N.A.		N.A.	Four-point bending	Loading to failure 0.1 mm/s	Max. bending moment ±SD (100.4 ± 38.5 Nm) Max. displt ± SD (2.7 ± 0.8 mm)
Tomaszewski et al. (2013)	Strain gauges	Human cadaveric femur	N.A.	N.A.	N.A.	N.A.		N.A.	1) heel strike (25% gait cycle)	1) F = 805 N	High stress concentration in the bone region close to the proximal end of the implant (Typical Gruen zones 3 and 5)
									2) toe off (55% gait cycle)	2) F = 720 N	
									3) one leg stance	3) F = 800 N	
Tomaszewski et al. (2010)	Generic *in silico* model	Human femur	From CT scan (slice thickness 3 mm)	E derived from *ρ*. Cortical bone E = 10,200 *ρ*. trabecular bone E = 33,900 *ρ*. Transition E = 5,307p + 469. v = 0.35	- ISP stem (cob-chro-mol) E = 210 GPa, ISP layer (spongiosa metal) E = 1 GPa, - OPRA E = 110 GPa. v = 0.3	Friction coefficient = 0.4 at first. Then implant and bone bonded		N.A.	1) heel strike (25% gait cycle)	1) Fr = 787 N, Mr = 32 Nm	High stress concentration in the bone region close to the proximal end of the implant (Typical Gruen zones 3 and 5)
									2) toe off (55% gait cycle)	2) Fr = 220 N, Mr = 38 Nm	
Tomaszewski et al. (2012b)	Generic *in silico* model	Human femur	From CT scan (slice thickness 3 mm) T score 0.1	E derived from *ρ*. Cortical bone E = 10,200 *ρ* trabecular bone E = 33,900 *ρ*. Transition E = 5,307p + 469 v = 0.4	- ISP stem (cobalt-chro alloy) E = 210 GPa, - Ti6Al4V stem E = 114 GPa, - OPRA E = 110 GPa. v = 0.3	friction coefficient. between stem and sleeve = 0.1. Implant and bone were bonded		N.A.	1) heel strike (25% gait cycle)	1) Fr = 787 N, Mr = 32 Nm	High stress concentration in the proximal region (Gruen zones 3 and 5). A stress peak was found in the region where the collar was in contact with the bone
									2) toe off (55% gait cycle)	2) Fr = 220 N, Mr = 38 Nm	
									3) forward fall loading	3) Fr = 678 N, Mr = 103 Nm	
Xu et al. (2006)	Generic *in silico* model	Human femur	From CT scan	Transversely Isotropic Longitudinal E = 18 GPa Radial and circumferential E = 13 GPa G = 7 GPa v = 0.3	Titanium stem E = 115 GPa, v = 0.30	Implant and bone bonded		N.A.	heel strike (25% gait cycle)	Fr = 3,750 N Mr = 75 Nm	Maximum stress was found at the proximal part of the femur (41.3 MPa)
Helgason et al. (2009)	Generic *in silico* model	Human femur	From CT scan	Isotropic E derived from *ρ*. E = 12.45 *ρ* GPa. v = 0.3	Ti6Al4V stem E = 110 GPa, v = 0.3	Implant and bone bonded		N.A.	Entire gait cycle	Fr = 708 N Mr = 39 Nm	High Von Mises stresses in bone (61.474 MPa) were found in the proximal part of the femur (Typical Gruen zones 3 and 5)
Lee et al. (2008b)	Generic *in silico* model	Sawbones (3rd gen)	Geometry reconstructed from the Sawbones femur	Transversely isotropic longitudinal E = 17 GPa shear modulus = 11.5 GPa Radial and circumferential moduli = 3.28 GPa	Titanium stem E = 115 GPa, v = 0.3	Implant and bone bonded		N.A.	1) Axial only force	1.1) 200 N 1.2) 400 N 1.3) 900 N 2.1) Fr = 866 N Mr = 14 Nm 2.2) Fr = 382 N Mr = 6 Nm 2.3) Fr = 192 N Mr = 3 Nm	Peak von Mises stresses in all the three loading cases were found at the proximal end of the implanted region. The peak stresses in Loading B in the implanted region were approximately three times higher
									2) F and M on the three axes when the subject transferred BW against a weigh scale at magnitudes of 200 N, 400 N, and 900 N		
									3) toe off (55% gait cycle)		
Ming, Xiang, and Yubo (2005)	Generic *in silico* model	N.A.	Geometry from standardized femur data	Transversely isotropic E_X_ = 11.50 GPa, E_Y_ = 11.50 GPa, E_Z_ = 17.00 GPa, v_XY_ = 0.58, v_YZ_ = 0.31, v_XZ_ = 0.31, G_XY_ = 3.60 GPa, G_YZ_ = 3.28 GPa, G_XZ_ = 3.28 GPa	Titanium stem E = 110 GPa, v = 0.3	Friction coefficient		N.A.	Entire gait cycle	F = 1,610 N	High von Mises stress in the model with contact interface (24 MPa)
						0.2				M = 54 Nm	
						- 0.5					
						- 1.0					
						- 2.0					

Note: E = Young’s Modulus, G = Shear modulus, v = Poisson’s ratio, *ρ* = ash density, Fr= Resultant Force, Mr = Resultant Moment.

Column 2 indicates the tools used to assess the stress concentration. Column 3 to 7 indicate the characteristics of the *in silico* model or *in vitro* experimental tests, while in the following columns were summarized the loading scenario and the testing protocol. The last one summarizes the main outcomes.

#### 3.3.2 Tools

In the *in vitro* experiments, both composite femur (Welke et al., 2013) and human cadaveric ones (Tomaszewski et al., 2013) were used. In the four-point-bending experiment, the force, the displacement, and the deflection in the mid-diaphysis were measured with a hydraulic material testing system and LVDT. This allowed estimating the stiffness, and the maximum moment at failure (Welke et al., 2013). Strain gauges were used to measure the strain magnitude at selected locations on the femur surface (Tomaszewski et al., 2013).

In the *in silico* simulations, Tomaszewski et al. derived the Young’s moduli of the bone elements from ash densities (Tomaszewski et al., 2010; Tomaszewski et al., 2012b), while (Ming, Xiang, and Yubo, 2005; Xu et al., 2006; Lee, et al., 2008b; Helgason et al., 2009; Newcombe et al., 2013) considered the Young’s moduli of the bone elements homogeneous (Ming, Xiang, and Yubo, 2005; Tomaszewski et al., 2010). used a coefficient of friction in a range between 0.2 and 2.0, which models the direct post-operative case, while in other cases, a fully bonded interface was simulated (Xu et al., 2006; Lee, et al., 2008b; Helgason et al., 2009; Tomaszewski et al., 2012a; Newcombe et al., 2013). Periprosthetic bone failure risk was evaluated based on the von Mises stress criterion in all the *in silico* simulations.

## 4 Discussion

Transfemoral osseointegrated prostheses might be an alternative to the socket prostheses, which represent the current standard-of-care. In fact, cost-benefit analyses reported that osseointegrated prostheses provide a better quality of life (in terms of physical functioning and bodily pain) at similar costs when compared to the socket prostheses (Haggstrom, Hansson, and Hagberg, 2013; Frossard et al., 2017; Black et al., 2022). However, like other uncemented prostheses, post-operative complications may occur due to lack of primary stability, stress-shielding and stress-concentration. The investigation of these failure scenarios is necessary for the preclinical assessment of transfemoral osseointegrated prostheses. However, the methods to investigate such failure scenarios, are far from consolidated: quite different methods and different criteria and metrics are reported in the literature. For these reasons, results are difficult to compare or even conflicting. Thus, the aim of this review was to summarize and compare the different methods used to assess the primary stability, stress shielding, and stress concentration of the osseointegrated lower limb prostheses.

### 4.1 Primary stability

The primary stability of the implant has been evaluated *in vitro* either measuring the extraction force, or the micromotions at the interface between the implant and the host bone. The extraction force was evaluated in the literature with two different methods: either with a pull-out or a push-out test. The pull-out test allows to evaluate the extraction force preserving the natural anatomy of the implanted femur (Welke et al., 2013; Jeyapalina et al., 2014). Conversely, the push-out test is usually performed on short segments resected from the femur, and is suitable if a short implant must be tested (Barnes et al., 2019). The push-out test allows to evaluate the extraction force of different stems on the same specimen in different osteotomy levels. However, the pull-out and push-out tests, which are used when a fast and reproducible test is needed, do not represent a common failure scenario for osseointegrated prostheses. In fact, the load is applied with a monotonic axial ramp until the exit of the prosthesis or the fracture of the specimen. However, the pull-out test is often used in other prosthetic devices as an FDA test (U.S. Department of Health and Human Services et al., 2003).

As aseptic loosening of osseointegrated prostheses is mainly driven by excessive micromotions under cyclic loading, a more representative pre-clinical assessment should quantify permanent migrations and/or inducible micromotions. The *in vitro* evaluation of the micromotion of the prosthesis was performed only by Barnes et al. using displacement transducers (LVDT) (Barnes et al., 2019). Nevertheless, a single LVDT allows to measure the displacement only in one direction. This limitation can be partially overcome, using multiple LVDTs to detect three-dimensional motions; however also in this case measurement errors may arise due to misalignment of the transducers. A further improvement is provided by DIC, which allows a full-field three-dimensional displacement analysis. In the literature it is possible to find similar studies with DIC on different devices (Morosato et al., 2020).

The micromotion can also be investigated with numerical models. In fact, once an *in silico* model has been developed and validated, it can be advantageously used to simulate and evaluate several complex loading scenarios and motor tasks, to compare different conditions such as different degrees of osseointegration, or modified bone quality (Martelli et al., 2011). In fact, numerical models give the opportunity to simulate experimental tests and repeat the same experiment a limitless number of times on the same models for an almost infinite variation of parameters (Romero et al., 2007; Martelli et al., 2011). Prochor et al. exploited the advantages of numerical models to explore the effect of different parameters such as implant length, implant diameter and interface bonding on the micromotions in a simplified load condition (Prochor and Anna Mierzejewska, 2019). A very important caveat is that numerical models should not be trusted if they are not thoroughly verified and validated, especially when making clinically relevant decisions (Cristofolini et al., 2010; ASME V&V 40, 2018; Oefner et al., 2021).

### 4.2 Strain-stress shielding

As bone remodelling is driven by frequently recurring cyclic loads, a commonly used method to evaluate the strain-stress shielding is simulating daily motor task. *In vitro* methodologies allow to apply controlled loading components. The strain on the surface of the specimens were evaluated using strain gauges, obtaining precise and accurate measurements on selected points (Cristofolini et al., 1998; Tomaszewski et al., 2013; Ahmed et al., 2020). DIC provides a full field strain analysis on the surface of the specimens and has already been used in literature (Palanca, Tozzi, and Cristofolini, 2016; Palanca et al., 2018).

However, *in vitro* mechanical tests allow to simulate only a limited set of loading conditions, while *in silico* methodologies allow to simulate different motor task and to investigate stress and strain at the interface between the prosthesis and the bone (Cristofolini et al., 2010). Moreover, the osseointegration between the prosthesis and the bone cannot be simulated *in vitro*, while with *in silico* models the different degree of osseointegration can be chosen, from unbonded with a given coefficient of friction (Tomaszewski et al., 2010; Prochor and Anna Mierzejewska, 2019; Ahmed et al., 2020) to fully bonded with a perfect osseointegration (Xu et al., 2006; Tomaszewski et al., 2012a; Tomaszewski et al., 2012b; Newcombe et al., 2013; Prochor and Sajewicz, 2018). All the numerical studies of this review are similar for what concerns the loading scenarios, while the quality of the bone tissue was represented with different degrees of detail. Indeed, some studies considered bone as isotropic (Tomaszewski et al., 2012a; Tomaszewski et al., 2012b; Prochor and Sajewicz, 2018; Prochor and Anna Mierzejewska, 2019), others anisotropic (Xu et al., 2006; Newcombe et al., 2013; Stenlund et al., 2017; Ahmed et al., 2020). In addition, some models considered the elastic modulus of the bone homogeneous (Xu et al., 2006; Newcombe et al., 2013; Stenlund et al., 2017; Prochor and Sajewicz, 2018; Prochor and Anna Mierzejewska, 2019; Ahmed et al., 2020), while others adapted the elastic modulus based on bone density (Tomaszewski et al., 2010; Tomaszewski et al. 2012a; Tomaszewski et al. 2012b). In a specific case, the bone was idealized with a cylindrical geometry (Stenlund et al., 2017) and with a generic geometry from standardized femur data (Prochor and Sajewicz, 2018), while in other cases the *in silico* model of the bone was based on computed tomography (CT) data of a femoral bone (Xu et al., 2006; Tomaszewski et al., 2010; Tomaszewski et al. 2012a; Tomaszewski et al. 2012b; Newcombe et al., 2013; Prochor and Anna Mierzejewska, 2019). However, bone density of some models was re-scaled to the typical young amputated patient since the CT images were *of* a cadaver old subject (Tomaszewski et al., 2010; Tomaszewski et al. 2012a; Tomaszewski et al. 2012b). Although different modelling strategies can be observed, comparable findings were obtained from different studies. This shows that the macro effects were captured by the models.

A limitation of these numerical studies is that in most cases the *in silico* models have not been experimentally validated. Indeed, Ahmed et al. and Tomaszewski et al. are among the few who used a validated model (Tomaszewski et al., 2013; Ahmed et al., 2020).

### 4.3 Strain-stress concentration

Evaluation of possible stress concentrations is crucial, as they can lead to bone fracture and subsequent implant failure. A commonly used method to evaluate the strain-stress concentration is simulating physiological loading, and the typical falling scenarios. Welke et al. (2013) aimed to investigate the ultimate load and the fracture modes with a simplified *in vitro* test. Typically, bending moment is the most critical loading scenario during normal daily activities, and a frequent cause for bone fractures. Thus, Welke et al. (2013) simulated the most critical condition by analyzing a highly repeatable loading scenario, i.e., the four-point-bending test. It must be noted that the four-point-bending test does not correspond to any realistic scenarios during the physiological conditions or falling. However, this testing condition may provide a first estimate of the load that can lead to a stress concentration and resulting in a bone fracture.

Subsequent studies evaluated the stress concentration around the implant using numerical simulations (Ming, Xiang, and Yubo, 2005; Xu et al., 2006; Lee, et al., 2008b; Helgason et al., 2009; Tomaszewski et al., 2010; Tomaszewski et al., 2012b; Newcombe et al., 2013). All the numerical models focusing on stress concentration are similar in loading strategies, while the quality of the bone tissue was represented with different degrees of detail. Indeed, some studies considered bone as isotropic (Helgason et al., 2009; Tomaszewski et al., 2010; Tomaszewski et al., 2012a), others anisotropic (Ming, Xiang, and Yubo, 2005; Xu et al., 2006; Lee, et al., 2008b; Newcombe et al., 2013). Some models applied homogeneous properties (Ming, Xiang, and Yubo, 2005; Xu et al., 2006; Lee, et al., 2008b; Helgason et al., 2009), while others modified the elastic modulus based on bone density (Tomaszewski et al., 2010; Tomaszewski et al., 2012b). In a specific case, the bone was idealized with a generic standardized geometry (Ming, Xiang, and Yubo, 2005), while in other cases the FE geometry of the bone was based on computed tomography (CT) scan of a femoral cadaveric bone (Xu et al., 2006; Helgason et al., 2009; Tomaszewski et al., 2010; Tomaszewski et al. 2012a; Tomaszewski et al. 2012b; Newcombe et al., 2013) or a femoral composite bone (Lee, et al., 2008b). However, as these models were based on a CT scan of an old subject, the bone density was re-scaled to the density that can be expected in amputees (typically young patients). Despite these different modelling strategies, comparable findings were obtained from the different studies showing that the macro effects were captured by the models.

*In silico* models estimating the risk of periprosthetic bone fractures mostly rely on the Von Mises stress criterion. The Von Mises criterion combines stress components and is applicable to ductile materials. Recent advances demonstrated that criteria based on the maximum principal strain are more accurate in predicting bone failure (Schileo et al., 2008; Miles et al., 2015).

Another limitation of many of these numerical studies is that *in silico* models have not been experimentally validated. To the authors’ knowledge, only Tomaszewski et al. reported a validated model (Tomaszewski et al., 2010; Tomaszewski et al., 2012a).

## 5 Conclusion

This systematic review highlighted and critically evaluated the main methods used to assess the primary stability, the stress-shielding and stress concentration of osseointegrated transfemoral prostheses, both through *in vitro* tests and *in silico* models. In some cases, results are difficult to compare as different methods were used to investigate the prostheses performance. It would be important to reach a consensus about the loading scenarios to be simulated, to enable comparison between studies. Furthermore, the use of a more versatile measurement systems, such as DIC, could be useful to develop a comprehensive method for *in vitro* testing the primary stability, the stress-shielding and stress concentration of an osseointegrated transfemoral prosthesis. The *in silico* models are generally more consistent in terms of loading scenarios, whereas different strategies are adopted to model the bone itself. More efforts should be dedicated to developing robustly validated *in silico* models. Moreover, *in vitro* and *in silico* methods can be synergistic, in order to provide more detailed and more reliable results than can be achieved with either approach singularly.
